# Based on the TLR4/NLRP3 Pathway and Its Impact on the Formation of NETs to Explore the Mechanism of Ginsenoside Rg_1_ on Acute Gouty Arthritis

**DOI:** 10.3390/ijms26094233

**Published:** 2025-04-29

**Authors:** Zhiman Li, Yang Yu, Qiang Sun, Zhilong Li, Xiaohui Huo, Jiyue Sha, Di Qu, Yinshi Sun

**Affiliations:** 1 Institute of Special Animal and Plant Sciences, Chinese Academy of Agricultural Sciences, Changchun 130112, China or lizm@caas.cn (Z.L.); 15941105393@163.com (Y.Y.); xiaohui.144@163.com (X.H.); s18946576631@163.com (J.S.); qudi@caas.cn (D.Q.); 2 College of Chinese Medicinal Materials, Jilin Agricultural University, Changchun 130118, China; sunqiang133543@163.com; 3 School of Pharmacy and Bioengineering, Chongqing University of Technology, Chongqing 401300, China; 13234437598@163.com

**Keywords:** ginsenoside Rg_1_, acute gouty arthritis, neutrophil extracellular traps, inflammatory response

## Abstract

This study investigated whether ginsenoside Rg_1_ (G-Rg_1_) alleviated acute gouty arthritis (AGA) in rats by modulating the TLR4/NLRP3 pathway and neutrophil extracellular trap (NET) formation. Rats were orally administered G-Rg_1_ or colchicine (Col) for 7 days, and monosodium urate (MSU) was injected into the ankle joints on day 5 to induce AGA. Joint swelling, histopathology (HE staining), and serum markers (MPO, NE, MPO-DNA, IL-6, IL-1β; ELISA) were assessed at the baseline and 6–36 h post-modeling. Western blot and immunofluorescence analyzed the NET-related and TLR4/NLRP3 pathway proteins in synovial tissue. G-Rg_1_ significantly reduced ankle swelling and synovial inflammation compared with the AGA group, lowered the serum IL-6, IL-1β, MPO, NE, and MPO-DNA levels, and suppressed NET-associated protein expression. Mechanistically, G-Rg_1_ downregulated TLR4/NLRP3 pathway activation in synovial tissue. These findings suggest that G-Rg_1_ mitigates AGA by inhibiting TLR4/NLRP3 signaling, thereby reducing inflammatory cytokine release and NET formation.

## 1. Introduction

Acute gouty arthritis (AGA) is a form of inflammatory arthritis primarily triggered by the deposition of monosodium urate (MSU) crystals [1]. The accumulation of MSU crystals in joint tissues promotes the release of inflammatory cytokines and the infiltration of inflammatory cells, thereby eliciting a specific inflammatory response in surrounding tissues. Clinically, this is characterized by redness, swelling, heat, and pain in the affected joints [2]. Over the past few decades, the global prevalence of gout has increased significantly. Epidemiological studies indicate that the global prevalence of gout ranges from 1% to 4%, imposing a significant disease burden worldwide [3]. The inflammatory response in AGA is primarily initiated by the interaction between MSU crystals and macrophages, resulting in the activation and assembly of the NLRP3 inflammasome [4]. The NLRP3 inflammasome is a cytoplasmic multiprotein complex that plays a pivotal role in the innate immune system by mediating the response to microbial infections. Its activation is dependent on a two-signal initiation system. The first signal entails the stimulation of NF-κB via TLR4 and TLR2, in conjunction with their shared adaptor protein MyD88, leading to the synthesis of pro-IL-1β and inflammasome components [5,6]. MSU crystals serve as the second signal, inducing the assembly of the NLRP3 inflammasome and the activation of caspase-1, thereby initiating downstream cascades. Caspase-1 cleaves pro-IL-1β into mature IL-1β [7]. Furthermore, the MAPK signaling pathway has been shown to play a role in the activation of the NLRP3 inflammasome [8]. MAPKs, which belong to a family of serine/threonine kinases, mediate the transmission of extracellular growth signals to the nucleus, thereby facilitating cell proliferation. These kinases are regarded as pivotal regulators of inflammatory responses and innate immunity [9]. The mammalian MAPK family includes several kinases such as extracellular signal-regulated kinases (ERK), p38, and c-Jun N-terminal kinases (JNK). These kinases are intimately linked to the NF-κB signaling pathway and are crucial for the activation of the NLRP3 inflammasome [8,10,11]. MSU crystals are capable of significantly activating the MAPK signaling pathway, as demonstrated by the upregulation of phosphorylated p38, ERK1/2, and JNK1/2/3 [12], thereby promoting activation of the NLRP3 inflammasome [13]. Following maturation, IL-1β binds to the IL-1β receptor, triggering downstream signaling cascades that result in the release of pro-inflammatory cytokines and chemokines. This process leads to the recruitment of neutrophils and other immune cells to the sites of crystal deposition [14]. Neutrophils, which are a critical component of the human immune system, rapidly migrate to sites of tissue injury following pathogen invasion, where they eliminate invading microorganisms through phagocytosis and degranulation. Activated neutrophils further contribute to pathogen containment and killing through the release of neutrophil extracellular traps (NETs) [15,16]. MSU crystals are capable of inducing inflammatory responses, thereby promoting NET formation. Moreover, NETs play a regulatory role in inflammation by degrading cytokines and chemokines [17]. Thus, we propose that targeting NETs effectively holds significant therapeutic potential for AGA. Additionally, complex interactions among the NLRP3 inflammasome, MAPK signaling pathway, IL-1β, and NETs may underlie the pathogenesis and progression of AGA.

Currently, the primary drugs employed in clinical practice for gout treatment include allopurinol, febuxostat, benzbromarone, and colchicine. Although these drugs exhibit anti-inflammatory and analgesic effects, most are linked to renal and hepatic toxicity, and long-term use may result in adverse effects including gastrointestinal damage [18]. Consequently, the development of safer and more effective therapeutic agents for gouty arthritis has emerged as a key research focus. Ginsenoside Rg_1_ (G-Rg_1_), a monomeric saponin derived from Panax ginseng, has demonstrated antioxidant, anti-inflammatory, and anti-apoptotic properties [19,20]. G-Rg_1_ has been shown to mitigate cardiomyocyte apoptosis and inflammation through the downregulation of LPS-induced inflammatory cytokine expression and the suppression of TLR4, NF-κB, and NLRP3 signaling [20]. Furthermore, G-Rg_1_ inhibits the expression of phosphorylated p38 MAPK and attenuates NLRP3 inflammasome activation as well as the IL-1β levels, thereby alleviating intestinal tight junction disruption in pigs [21]. Given that AGA activates the NLRP3 inflammasome via TLR4 and MAPK signaling to induce an inflammatory response, and considering that the release of neutrophil extracellular traps (NETs) is intimately associated with the inflammatory response [22], this study aimed to elucidate the mechanism of G-Rg_1_ in treating AGA by examining its impact on NET formation mediated by the TLR4/NLRP3 pathway. This study aims to provide an experimental foundation for the clinical application of G-Rg_1_.

## 2. Results

### 2.1. Effects of G-Rg_1_ on Ankle Joint Pathology in AGA Rats

**Figure 1:**
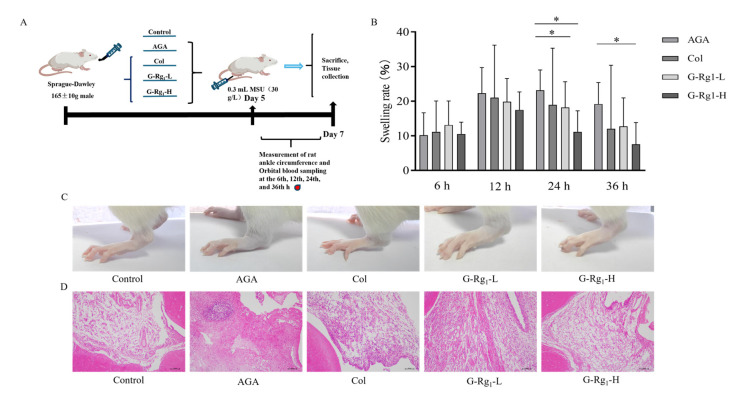
Effects of G-Rg_1_ on ankle joint pathology in AGA rats. (**A**) Overall test protocol for the ameliorative effect of G-Rg_1_ in AGA rats. (**B**) Effects of G-Rg_1_ on the rate of ankle swelling in rats. (**C**) Effects of G-Rg_1_ on the degree of ankle swelling in rats. (**D**) Effects of G-Rg_1_ on histopathologic changes in the synovial membrane of rat ankle joints (×100). Note: Compared with the AGA group, * *p* < 0.05.

The ankle circumference of the rats was measured, and the results demonstrated that the ankle swelling in the AGA group was significantly higher than that in the control group, confirming the successful establishment of the AGA model. The ankle swelling in the AGA group peaked at 24 h and was significantly higher than that in the G-Rg_1_-L and G-Rg_1_-H groups (*p* < 0.05). Rats treated with G-Rg_1_ showed a significant reduction in swelling at 24 h compared with the AGA group (*p* < 0.05). At 36 h, the ankle joint swelling rate in the AGA group remained significantly higher than that in the G-Rg_1_-H group (*p* < 0.05) (Figure 1B). Observation of the ankle joints revealed that compared with the control group, the AGA group exhibited severe swelling, loss of bony markings, curled and weakened foot paws, and impaired mobility. In contrast, rats treated with G-Rg_1_ (12.5 mg/kg, 25 mg/kg) for 36 h showed a significant reduction in swelling and a marked alleviation of symptoms (Figure 1C). Histopathological analysis of the rat ankle synovial tissue by HE staining (Figure 1D) revealed that the control group exhibited clear cell structures, sparse and orderly arrangement, and no signs of inflammatory cell infiltration or tissue hyperplasia. In the AGA group, the synovial tissue exhibited marked hyperplasia, extensive inflammatory cell infiltration, lymphocyte clustering, and prominent fibrous tissue hyperplasia, confirming the successful induction of the AGA model. In the Col, G-Rg_1_-L, and G-Rg_1_-H groups, the inflammatory cell infiltration was significantly reduced, fibrous tissue proliferation was attenuated, and cell arrangement was more organized compared with the AGA group. These results demonstrate that G-Rg_1_ effectively inhibited ankle joint swelling and ameliorated the histopathological morphology of the synovium in AGA rats.

### 2.2. Effects of G-Rg_1_ on Serum Inflammatory Factors in AGA Rats

**Figure 2:**
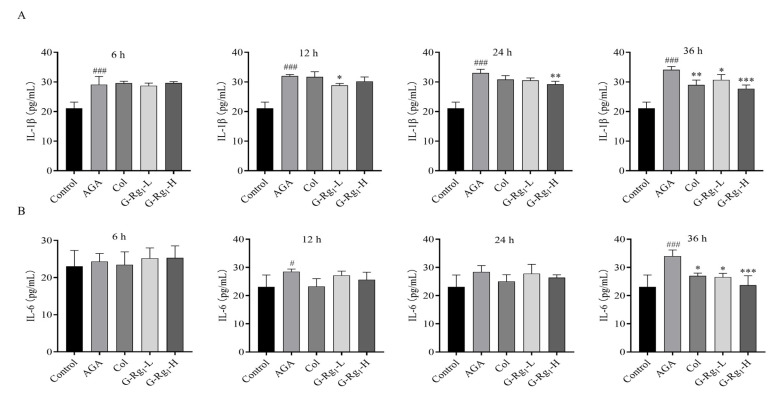
Effects of G-Rg_1_ on serum inflammatory factors in AGA rats. (**A**) Effects of G-Rg_1_ on IL-1β in rat serum at different time points. (**B**) Effects of G-Rg_1_ on IL-6 in rat serum at different time points. Note: Compared with the control group, ^#^ *p* < 0.05, ^###^ *p* < 0.001; compared with the AGA group, * *p* < 0.05, ** *p* < 0.01, *** *p* < 0.001.

The serum inflammatory factors in AGA rats were measured, and the results demonstrated that the serum IL-1β levels were significantly higher in the AGA group compared with the control group at all time points (*p* < 0.001). The G-Rg_1_-L group showed significantly lower IL-1β levels than the AGA group at 12 h (*p* < 0.05), and the G-Rg_1_-H group showed significantly lower IL-1β levels than the AGA group at 24 h (*p* < 0.01). At 36 h, IL-1β levels in both the colchicine and G-Rg_1_ (12.5 mg/kg, 25 mg/kg) treatment groups were significantly lower than those in the AGA group (*p* < 0.01, *p* < 0.05 and *p* < 0.001, respectively), with G-Rg_1_ exhibiting a concentration-dependent effect (Figure 2A). No significant differences in the IL-6 levels were observed among the groups at 6 h. However, the AGA group exhibited significantly higher IL-6 levels than the control group at 12 h and 36 h (*p* < 0.05 and *p* < 0.001, respectively). At 36 h, IL-6 levels in the Col, G-Rg_1_-L, and G-Rg_1_-H groups were significantly lower than those in the AGA group (*p* < 0.05 and *p* < 0.001), with G-Rg_1_ demonstrating a concentration-dependent ameliorative effect (Figure 2B). These findings indicate that G-Rg_1_ effectively reduced inflammatory factors in the serum of AGA rats.

### 2.3. Effects of G-Rg_1_ on NETs Markers in the Serum of AGA Rats

NETs are composed of a DNA backbone embedded with various proteins, including MPO, NE, histones (primarily citrullinated histone 3, CitH3), antimicrobial peptides (LL-37), and serine proteases, among others. These components collectively mediate the antimicrobial and pro-inflammatory functions of NETs [23]. The MPO-DNA complex, an extracellular protein colocalization product, is widely used for the detection of NETs due to its high specificity [24]. To investigate the effect of G-Rg_1_ on NETs formation in AGA rats, the MPO activity, NE activity, and MPO-DNA complex levels were measured in the serum at various time points post-modeling.

**Figure 3:**
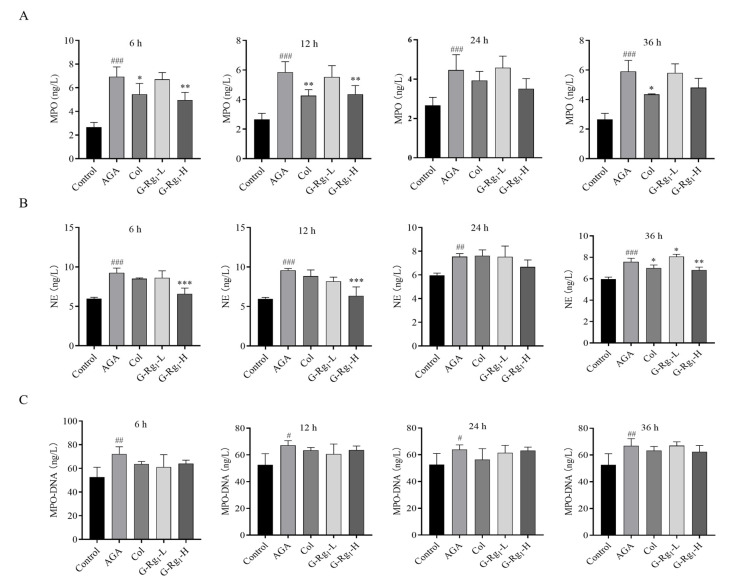
Effects of G-Rg_1_ on NET-related indices in the serum of AGA rats. (**A**) Effects of G-Rg_1_ on MPO levels in the serum of AGA rats at different time points. (**B**) Effects of G-Rg_1_ on NE levels in the serum of AGA rats at different time points. (**C**) Effects of G-Rg_1_ on MPO-DNA content in the serum of AGA rats at different time points. Note: Compared with the control group, ^#^ *p* < 0.05, ^##^ *p* < 0.01, ^###^ *p* < 0.001; compared with the AGA group, * *p* < 0.05, ** *p* < 0.01, *** *p* < 0.001.

MPO activity in the AGA group was significantly higher than that in the control group at all time points (*p* < 0.005). MPO activity in the Col group was significantly lower than that in the AGA group at 6 h, 12 h, and 36 h (*p* < 0.05, *p* < 0.01), Additionally, MPO activity in the G-Rg_1_-H group was markedly lower than that in the AGA group at 6 h and 12 h (*p* < 0.01) (Figure 3A). NE activity in the AGA group was significantly elevated compared with the control group at 6 h, 12 h, 24 h, and 36 h (*p* < 0.001, *p* < 0.01). In contrast, NE activity in the G-Rg_1_-H group was significantly reduced compared with the AGA group at 6 h, 12 h, and 36 h (*p* < 0.001, *p* < 0.01) (Figure 3B). The serum levels of the MPO-DNA complexes in the AGA group were significantly elevated compared with the control group at all time points (*p* < 0.01, *p* < 0.05). The MPO-DNA levels in the treated groups were consistently lower than those in the AGA group at all time points, though the differences did not reach statistical significance (Figure 3C). These findings suggest that G-Rg_1_ may mitigate the inflammatory response in AGA by suppressing NET release.

### 2.4. Effects of G-Rg_1_ on the Expression of NETs-Related Proteins in Synovial Tissues of AGA Rats

**Figure 4:**
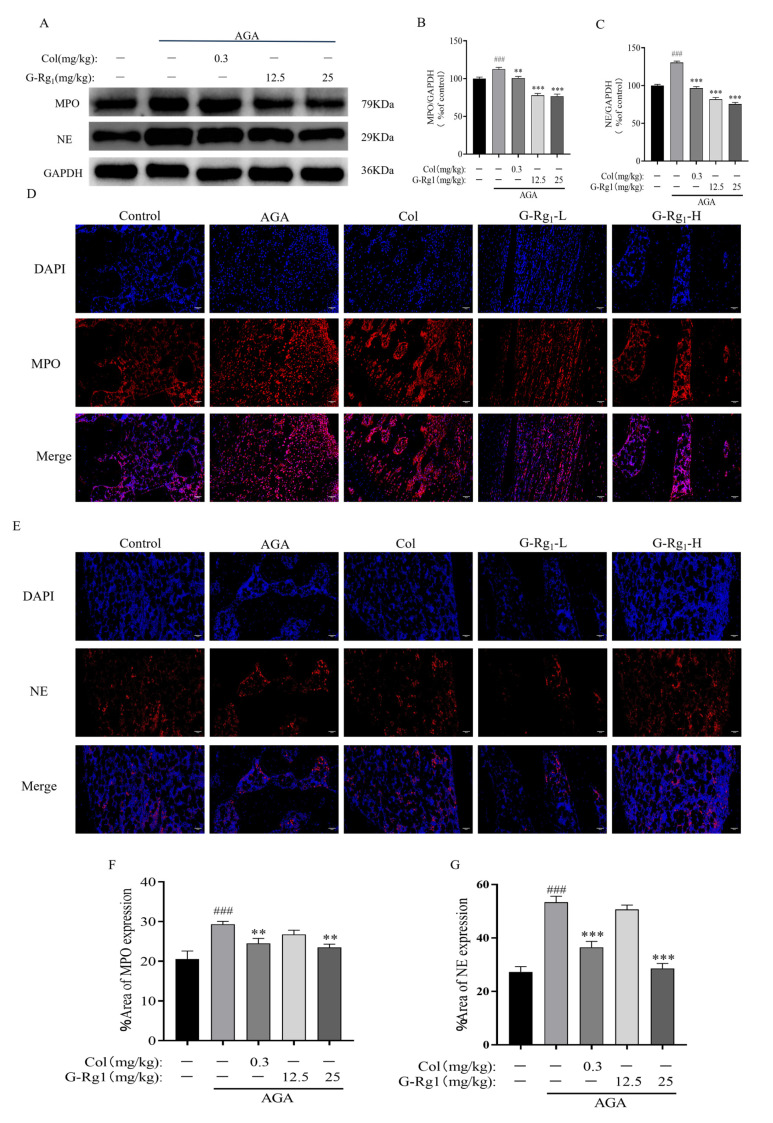
Effects of G-Rg_1_ on the expression of NET-related proteins in the synovial tissues of the ankle joints of AGA rats. (**A**) Representative bands of MPO and NE protein expression in the synovial tissues of the ankle joints of AGA rats measured by Western blot. (**B**) Protein expression of MPO. (**C**) Protein expression of NE. (**D**) Expression of MPO in the synovial tissues of the ankle joint of AGA rats measured by immunofluorescence (×200). (**E**) Expression of NE in the synovial tissues of the ankle joint of AGA rats measured by immunofluorescence (×200). (**F**) MPO immunofluorescence intensity. (**G**) NE immunofluorescence intensity. Note: Compared with the control group, ^###^ *p* < 0.001; compared with the AGA group, ** *p* < 0.01, *** *p* < 0.001.

Immunofluorescence and Western blot analyses revealed that the expression levels of MPO and NE, key NETs-associated proteins, were significantly upregulated in the synovial tissues of the AGA group following MSU injection (*p* < 0.001) (Figure 4). These findings suggest enhanced NETs release and subsequent inflammatory activation in the synovial tissue of AGA rats. Following drug intervention, the expression levels of MPO and NE were significantly reduced in the Col, G-Rg_1_-L, and G-Rg_1_-H groups compared with the AGA group (*p* < 0.01, *p* < 0.001). These results demonstrate that G-Rg_1_ effectively suppresses the inflammatory response mediated by NETs formation and release, thereby exerting anti-inflammatory effects in AGA treatment.

### 2.5. Effects of G-Rg_1_ on Protein Expression of the TLR4/NLRP3 Pathway in Synovial Tissues of AGA Rats

**Figure 5:**
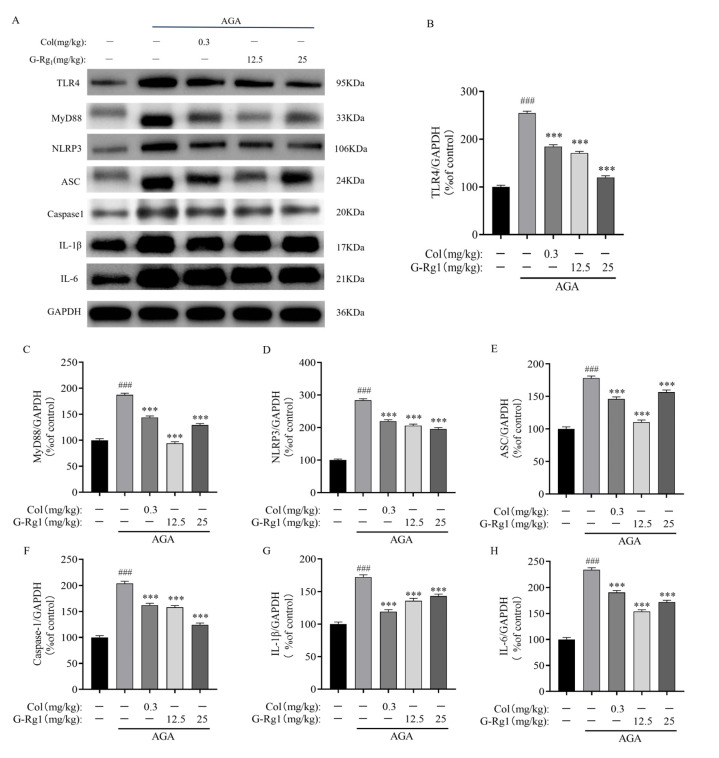
Effects of G-Rg_1_ on protein expression of TLR4/NLRP3 pathway in synovial tissues of ankle joints of AGA rats. (**A**) Representative bands of TLR4/NLRP3 pathway protein expression in synovial tissues of ankle joints of AGA rats by western blot. (**B**) Protein expression of TLR4. (**C**) Protein expression of MyD88. (**D**) Protein expression of NLRP3. (**E**) Protein expression of ASC. (**F**) Protein expression of Caspase-1. (**G**) Protein expression of IL-1β. (**H**) Protein expression of IL-6. Note: Compared with the control group, ^###^ *p* < 0.001; compared with the AGA group, *** *p* < 0.001.

The protein expression levels of TLR4, MyD88, NLRP3, ASC, Caspase1, IL-1β, and IL-6 were significantly upregulated in the ankle joints of rats in the AGA group following MSU injection (*p* < 0.005). This indicates activation of the TLR4/NLRP3 signaling pathway and production of mature IL-1β, confirming the successful establishment of the AGA rat model. Compared with the AGA group, the expression levels of TLR4, MyD88, NLRP3, ASC, Caspase1, IL-1β, and IL-6 in the synovial tissues were significantly downregulated following treatment with Colchicine (0.3 mg/kg) and G-Rg_1_ (12.5 mg/kg, 25 mg/kg) (*p* < 0.005) (Figure 5). These results demonstrate that G-Rg_1_ effectively suppresses the TLR4/NLRP3 pathway, thereby exerting anti-inflammatory effects in AGA rats.

### 2.6. Effects of G-Rg_1_ on MAPK Pathway Protein Expression in Synovial Tissues of AGA Rats

**Figure 6:**
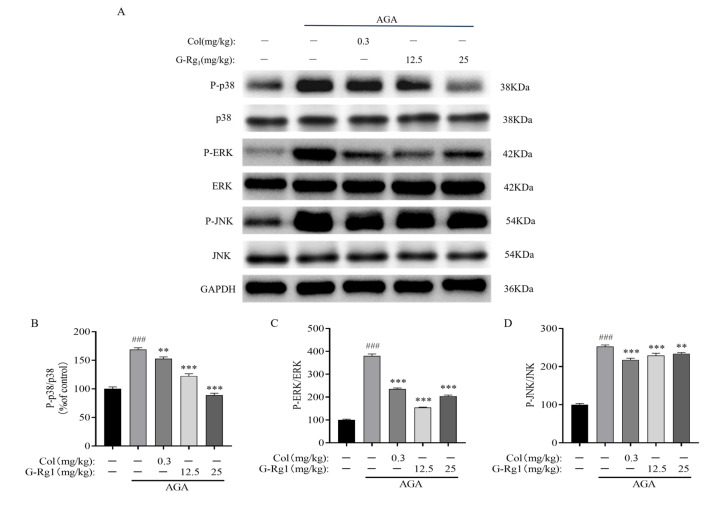
Effects of G-Rg_1_ on MAPK pathway protein expression in the synovial tissues of the ankle joints of AGA rats. (**A**) Representative bands of MAPK pathway protein expression in the synovial tissues of the ankle joints of AGA rats by Western blot. (**B**) Protein expression of P-p38/p38. (**C**) Protein expression of P-ERK/ERK. (**D**) Protein expression of P-JNK/JNK. Note: Compared with the control group, ^###^ *p* < 0.001; compared with the AGA group, ** *p* < 0.01, *** *p* < 0.001.

The phosphorylation levels of JNK, ERK, and p38 were significantly upregulated in the ankle joints of rats in the AGA group following MSU injection (*p* < 0.001). Compared with the AGA group, the phosphorylation levels of JNK, ERK, and p38 were significantly downregulated in the Col, G-Rg_1_-L, and G-Rg_1_-H groups (*p* < 0.01, *p* < 0.001) (Figure 6). These findings indicate that G-Rg_1_ effectively suppresses the MAPK pathway, thereby alleviating AGA symptoms and delaying disease progression.

## 3. Discussion

During the acute phase of gout, mature IL-1β induces the release of abundant NETs by neutrophils. NETs, via the exposure of their components, activate and recruit additional immune cells, thereby amplifying localized acute inflammatory responses [25]. Concurrently, the TLR4 and MAPK signaling pathways play pivotal roles in this process by activating the NLRP3 inflammasome, thereby promoting the release of IL-1β. The stimulation of NET formation via these pathways is intimately associated with the onset and progression of AGA. Collectively, these signaling pathways and cellular responses represent key mechanisms underlying the pathophysiology of AGA.

In our experimental model, intra-articular MSU crystal injection successfully induced AGA, as evidenced by progressive ankle swelling within 24 h, impaired gait, synovial hyperplasia, and inflammatory cell infiltration. Molecular analysis revealed a significant upregulation of TLR4, NLRP3, ASC, and caspase-1 in the synovial tissues, confirming TLR4-mediated NLRP3 inflammasome activation. Notably, the MyD88-dependent pathway, a key signaling cascade downstream of TLR4 activation [26], initiated a series of intricate intracellular signaling cascades upon activation. These responses not only involve the interaction of multiple molecules, but also profoundly influence cellular physiological functions, thereby playing a pivotal role in AGA pathogenesis. For MAPK, a critical regulator of inflammatory responses and innate immunity [8], Western blot analysis further demonstrated MAPK pathway activation in AGA rats, with elevated phosphorylation of p38, ERK, and JNK in the synovial tissues. This suggests that MSU crystals activate the MAPK signaling pathway in AGA rats. The activation of the MAPK signaling pathway further promoted NLRP3 inflammasome assembly and activation, enhanced the release of inflammatory mediators such as IL-1β, and exacerbated the inflammatory response, as demonstrated by elevated levels of IL-1β and IL-6 in the AGA group.

It is well-established that mature IL-1β binds to the IL-1β receptor, inducing immune cells to produce pro-inflammatory cytokines such as IL-1β and IL-6, which are critical for neutrophil recruitment [27]. Subsequently, recruited neutrophils release NETs, exposing their components to activate and recruit additional immune cells, thereby amplifying the local acute inflammatory response [25]. This is supported by the elevated serum levels of NET markers (MPO, NE, and MPO-DNA complexes) and the synovial tissue overexpression of MPO and NE in AGA rats. These findings demonstrate that MSU crystals induce an inflammatory response in the rat ankle joints, triggering NET release by neutrophils. This process, mediated by MSU crystal stimulation, activates and recruits immune cells, leading to localized inflammation, a key driver of gout onset. This finding not only enhances our understanding of gout pathogenesis, but also identifies a potential therapeutic target for gout treatment.

Following G-Rg_1_ administration, the ankle joint swelling in rats was significantly reduced, and the swelling rate remained consistently lower than that of the AGA group within 48 h. Furthermore, the swelling subsided more rapidly in the G-Rg_1_-treated group compared with the AGA group, and the rats exhibited restored walking gait. Inflammatory cell infiltration in the synovial tissue was also significantly reduced. Inflammatory cell infiltration, a hallmark of inflammatory diseases such as arthritis, was attenuated, indicating a reduction in the inflammatory response. Additionally, the synovial tissue exhibited a more organized cellular architecture compared with the AGA group, suggesting the restoration and normalization of tissue structure. At the molecular level, the expression of the TLR4 and MAPK signaling pathways in the synovial tissues was significantly downregulated in the G-Rg_1_-treated group compared with the AGA group, indicating that G-Rg_1_ effectively inhibits the MSU crystal-induced activation of TLR4 and MAPK signaling, suppresses NLRP3 inflammasome assembly and activation, and thereby attenuates the release of inflammatory factors. This is further supported by the significant reduction in IL-6 and IL-1β levels in the serum and synovial tissues of the G-Rg_1_-treated group at 36 h post-MSU injection compared with the AGA group, indicating effective control of the inflammatory response within 36 h. Notably, G-Rg_1_ treatment significantly decreased MPO and NE expression in the synovial tissues and serum, indicating the inhibition of NET formation. These results not only demonstrate the significant efficacy of G-Rg_1_ in modulating key inflammatory markers, but also suggest its role in regulating NET-mediated processes, including the capture and degradation of pro-inflammatory mediators, thereby promoting the resolution of neutrophil-driven inflammation. NETs, extracellular structures released by neutrophils in response to infection or injury, not only capture and kill pathogens, but also degrade pro-inflammatory mediators, thereby playing a critical role in the resolution of inflammation [28]. G-Rg_1_ modulates this process by inhibiting the formation and release of NETs, thereby achieving therapeutic effects in AGA.

## 4. Materials and Methods

### 4.1. Instruments and Reagents

Epo ch2 Microplate Reader (BioTek Instruments, Inc., Winooski, VT, USA); DHG-9123A Electrothermal Constant-Temperature Drying Oven (Shanghai Jinghong Experimental Equipment Co., Ltd., Shanghai, China); CPA225D Electrothermal Constant-Temperature Water Bath (Beijing Yongguangming Medical Instrument Factory, Beijing, China); MS204S Electronic Analytical Balance (Mettler Toledo, Columbus, OH, USA); Heraeus Megafuge 8R High-Speed Centrifuge (Thermo Fisher Scientific, Waltham, MA, USA); BX53 Inverted Fluorescence Microscope (Olympus Corporation, Tokyo, Japan); DYY-8C Electrophoresis Power Supply (Beijing Liuyi Instrument Factory, Beijing, China); DYCZ-40D Vertical Electrophoresis System (Beijing Liuyi Instrument Factory, Beijing, China); DYCZ-24DN Vertical Electrophoresis System (Beijing Liuyi Instrument Factory, Beijing, China); ChemiDoc™ MP Imaging System (Bio-Rad Laboratories, Inc., Hercules, CA, USA); KD-BM Tissue Embedding Station (Zhejiang Jinhua Kedi Instrument and Equipment Co., Ltd., Jinhua, China); KD3390 Microtome (Zhejiang Jinhua Kedi Instrument and Equipment Co., Ltd., Jinhua, China); KD-T1 Tissue Flotation and Baking System (Zhejiang Jinhua Kedi Instrument and Equipment Co., Ltd., Jinhua, China).

Colchicine and monosodium urate (MSU) were purchased from Sigma-Aldrich (Shanghai) Trading Co., Ltd. (Shanghai, China). Ginsenoside Rg_1_ (G-Rg_1_) was purchased from Ruifengfan Technology Co., Ltd. (Kaifeng, China). The Rat Myeloperoxidase (MPO) ELISA Kit, Rat Neutrophil Elastase (NE) ELISA Kit, Rat MPO-DNA Complex ELISA Kit, Rat Interleukin-6 (IL-6) ELISA Kit, and Rat Interleukin-1β (IL-1β) ELISA Kit were purchased from Jiangsu Feiya Biotechnology Co., Ltd. (Yancheng, China). RIPA lysis buffer was purchased from BioWorld. (Alachua, FL, USA). The BCA Protein Assay Kit was purchased from Beyotime Biotechnology Co., Ltd. (Shanghai, China). Glyceraldehyde-3-phosphate dehydrogenase (GAPDH) antibody and mouse anti-IgG antibody were purchased from Abcam (Cambridge, MA, USA). Extracellular signal-regulated kinase (ERK), c-Jun N-terminal kinase (JNK), p38 mitogen-activated protein kinase (p38), phosphorylated ERK (p-ERK), phosphorylated JNK (p-JNK), and phosphorylated p38 (p-p38) antibodies were purchased from Santa Cruz Biotechnology (Dallas, TX, USA). Myeloperoxidase (MPO), neutrophil elastase (NE), caspase-1, apoptosis-associated speck-like protein containing a CARD (ASC), NLR family pyrin domain containing 3 (NLRP3), myeloid differentiation primary response 88 (MyD88), and toll-like receptor 4 (TLR4) antibodies were purchased from Beijing Biosynthesis Biotechnology Co., Ltd. (Beijing, China).

### 4.2. Experimental Animals

Specific pathogen-free (SPF)-grade Sprague-Dawley (SD) healthy male rats, weighing 180 ± 10 g, were purchased from Liaoning Changsheng Biotechnology Co., Ltd. (Benxi, China) (Animal Production License: SCXK (Liao) 2022-0001). The rats were provided ad libitum access to food and water and allowed to acclimate to the rearing environment, which was maintained at an ambient temperature of 22 ± 2 °C, a relative humidity of 55% ± 15%, and a 12 h light/dark cycle.

### 4.3. Methods

#### 4.3.1. Animal Grouping and Establishment of the AGA Rat Model

The SD rats were randomly allocated into five groups: a control group (Control), a model group (AGA), a colchicine positive control group (Col, 0.3 mg/kg), a G-Rg_1_ low-dose group (G-Rg_1_-L, 12.5 mg/kg), and a G-Rg_1_ high-dose group (G-Rg_1_-H, 25 mg/kg), with five rats in each group. Rats in the treatment groups were orally administered the corresponding test substances once daily for 7 days. The control group was administered the same volume of saline via oral gavage under the same conditions. On day 5, AGA was induced by intra-articular injection of 0.3 mL of an MSU crystal suspension (30 g/L) into the bilateral ankle joint cavities of rats in the AGA, Col, G-Rg_1_-L, and G-Rg_1_-H groups. The control group received an equivalent volume of sterile saline under the same conditions. At five time points (baseline (before modeling) and at 6, 12, 24, and 36 h post-modeling), the ankle joint circumference (cm) of the rats was measured to evaluate joint swelling. Blood samples were collected from the retro-orbital plexus for further analysis.

#### 4.3.2. Measurement of Rat Ankle Joint Swelling Rate

The ankle joint circumference (cm) of the rats was measured at five time points: baseline (before modeling) and at 6, 12, 24, and 36 h post-modeling. The swelling rate of the ankle joint was calculated to assess the degree of joint swelling using the following equation: Swelling Rate (%) = (Post-modeling Ankle Circumference − Pre-modeling Ankle Circumference)/Pre-modeling Ankle Circumference × 100%.

#### 4.3.3. Histopathological and Morphological Analysis of Rat Ankle Synovial Tissue

The synovial tissue of the rat ankle joint was fixed in 4% paraformaldehyde, decalcified using 10% EDTA solution, processed for paraffin embedding, sectioned at a 5 μm thickness, and stained with hematoxylin and eosin (H&E). After dehydration, clearing, and mounting with neutral balsam, the histopathological and morphological changes in the synovial tissue were observed under a light microscope.

#### 4.3.4. Determination of NETs and Inflammatory Factors in Rat Serum

Blood samples were collected from the retro-orbital plexus of rats at five time points: baseline (before modeling) and at 6, 12, 24, and 36 h post-modeling. The serum levels of MPO, NE, and MPO-DNA complex, which are markers of NETs, as well as the inflammatory cytokines IL-6 and IL-1β, were measured. All procedures were performed in strict accordance with the manufacturer’s instructions to ensure standardized operations.

#### 4.3.5. Analysis of NETs-Related Proteins and TLR4/NLRP3 Pathway Protein Expression in Rat Ankle Synovial Tissues

Synovial tissue from the rat ankle joints was homogenized in 200 μL of RIPA lysis buffer supplemented with 2 μL of phenylmethylsulfonyl fluoride (PMSF) to extract total proteins. The supernatant was collected after centrifugation, and the protein concentration was determined using the bicinchoninic acid (BCA) assay. Equal amounts of protein samples were separated by sodium dodecyl sulfate-polyacrylamide gel electrophoresis (SDS-PAGE) using 8–12% resolving gels and transferred onto polyvinylidene fluoride (PVDF) membranes. The membranes were blocked with 5% (*w*/*v*) non-fat milk or 5% (*w*/*v*) bovine serum albumin (BSA) for 2 h at room temperature, followed by washing four times with phosphate-buffered saline containing 0.1% Tween-20 (PBST) (8 min each). The membranes were then incubated with specific primary antibodies at 4 °C overnight. After washing four times with PBST (8 min each), the membranes were incubated with horseradish peroxidase (HRP)-conjugated species-specific secondary antibodies for 2 h at room temperature. For signal detection, enhanced chemiluminescence (ECL) reagents A and B were mixed in equal volumes and applied to the PVDF membranes. The PVDF membrane was placed in full contact with the chemiluminescent substrate, and signals were captured using an imaging system. The band intensities were quantified using ImageJ software (version: 1.x), and the relative grayscale values were normalized to the internal control GAPDH. The relative protein expression levels were calculated, and data were subjected to statistical analysis to compare differences between groups.

#### 4.3.6. Detection of NETs in Rat Ankle Joint Synovial Tissue

Synovial tissue sections from rat ankle joints were deparaffinized, and the sections were put into beryllate buffer, incubated with fire for 20 min for antigen repair, washed with PBS 3 times, each time for 5 min; incubated with 3% H_2_O_2_ for 10 min, and washed with PBS 3 times, each time for 2 min; the primary antibody was added and incubated at 4 °C overnight. After warming at room temperature for 30 min, the secondary antibody was added and incubated at room temperature for 1 h. The sections were washed 3 times with PBS for 5 min each time, and then the nuclear DNA of the labeled cells was re-stained with DAPI staining solution, and the sections were incubated at room temperature for 10 min, and the images were captured by fluorescence microscope.

#### 4.3.7. Statistical Analysis

The experimental data are expressed as the mean ± standard error of the mean (SEM). Statistical analysis was performed using GraphPad Prism software (version 8.0.1; GraphPad Software, San Diego, CA, USA).

## 5. Conclusions

In summary, the results of this study demonstrate that G-Rg_1_ ameliorates arthropathic conditions and inflammatory responses in the AGA rat model. The mechanism of action likely involves the downregulation of TLR4/MyD88 and MAPK pathway-related proteins, thereby inhibiting NLRP3 inflammasome assembly and suppressing the release of inflammatory factors such as IL-1β and IL-6. This study not only elucidates the potential mechanism underlying G-Rg_1_’s anti-inflammatory effects in the AGA rat model but also provides a robust experimental foundation for its clinical application. These findings offer novel insights and directions for developing therapeutic agents targeting inflammatory diseases, such as gouty arthritis. However, despite the promising experimental results, the efficacy and safety of G-Rg_1_ in human diseases require validation through further clinical trials.

## Data Availability

Data will be made available on request.
